# The Incidental Resolution of Severe Alcohol Use Disorder during Esketamine Treatment of Major Depressive Disorder: A Case Report

**DOI:** 10.1155/2022/8992697

**Published:** 2022-12-15

**Authors:** Zeeshan Faruqui, Christopher Kim

**Affiliations:** ^1^Drexel University College of Medicine, 2900 W Queen Lane, Philadelphia, PA 19129, USA; ^2^Keystone Behavioral Health, 110 Chambers Hill Drive, Chambersburg, PA 17201, USA

## Abstract

Alcohol use disorder (AUD) is especially prevalent among individuals with major depressive disorder (MDD) and is associated with higher morbidity, mortality, disability, and risk for suicide. Intranasal esketamine has been documented to be a safe and effective option for treatment resistant MDD and received US FDA approval in 2019. However, the availability of esketamine is limited due to requirements for a Risk Evaluation and Mitigation Strategy program for its administration as well as concerns over substance use disorders (SUD) as a potential contraindication or adverse effect. This case presents a 61-year-old female with a history of severe recurrent treatment resistant MDD and severe AUD who has expressed interest in esketamine. To date, she has completed 20 sessions of 56 mg intranasal esketamine twice a week before increasing to 5 weekly sessions and then 14 weekly sessions of 84 mg. Although there was immediate subjective improvement in mood as well as reductions in PHQ-9 and HAM-D scores, the dosage was increased to extend the therapeutic benefit throughout the entire intertreatment interval. She was able to complete these sessions without complication. Incidentally, she also reported decreased desire to use alcohol, decreased impulsivity, and the complete cessation of alcohol use by the second week. While on 56 mg, she had a single relapse of binging which was addressed with motivational therapy and has remained alcohol-free since. This case presents the first documented example of the safe and effective use of intranasal esketamine in patient with treatment resistant MDD in which there was incidental resolution of their comorbid severe AUD. This suggests that esketamine can be used without risk of SUD onset or exacerbation. Thus, esketamine should not be discounted in patients with SUD and continued research is needed to further elucidate its role in treating SUD.

## 1. Introduction

Alcohol use disorder (AUD) is especially prevalent among individuals with major depressive disorder (MDD) and is associated with higher morbidity, mortality, disability, and risk for suicide [1–4]. The cooccurrence of these diseases result in a mutually destructive exacerbation of increased high-risk drinking and worse depressive symptoms. The current standard of care for MDD utilizes a combination of pharmacotherapy (i.e., SSRI or SNRI) and psychotherapy (i.e., CBT). A significant percentage of patients with MDD fail two or more monotherapies reclassifying them as treatment resistant MDD. Intranasal esketamine coadministered with an antidepressant has been well established as a safe and effective option for treatment resistant MDD and received United States (US) Food and Drug Administration approval in 2019 [5–8]. Esketamine has been found to be more potent and have less side effects in addition to having an easier route of administration than ketamine [9, 10]. Despite this, Esketamine availability is still limited and potentially underutilized among patients who could benefit from this treatment. Contributing factors could include the stringent certification criteria required for its use and calls for further research in determining ideal patient populations, contraindications, and long-term safety. Esketamine administration requires a Risk Evaluation and Mitigation Strategy program to manage adverse effects and to prevent diversion. This includes a certified medical clinic where prequalified patients must register and come into to self-administer the spray under supervision by a trained medical provider. Its main adverse effects include dissociation, dysgeusia, nausea, and hypertension. It is the hallucinogenic effect that gives esketamine street value as demonstrated by its status as a schedule III agent. Along with this potential misuse, it would not be unreasonable to hypothesize that esketamine use in a patient with an ongoing or history of substance use disorder (SUD) may be of greater risk to new onset or exacerbation of an existing SUD. However, there is no evidence of increased SUD from esketamine use [11]. Rather, there has been a recent spike in interest in esketamine as a potential treatment for SUD such as alcohol among some researchers and popular media. Although the current body of evidence does not support such an assertion, this case provides an example of its potential benefits [12].

This case report highlights the incidental resolution of severe AUD in a single patient with treatment resistant MDD while undergoing esketamine treatment. This unintended consequence was volunteered by the patient after just a few sessions and has been maintained to date except for a single episode of relapse by binging.

## 2. Case Presentation

The patient was a 61-year-old female nurse with a history of previously diagnosed severe recurrent treatment resistant MDD without psychotic features and severe AUD who came to the clinic expressing interest in ketamine after speaking with another patient. Her past medical history includes generalized anxiety disorder, essential hypertension, colon cancer, and breast cancer.

On presentation, she reported feeling depressed her whole life described as depressed mood, anhedonia, insomnia, feelings of guilt and worthlessness, low energy, decreased concentration, weight loss with decreased appetite, psychomotor agitation, and a passive death wish verbalized during intoxication satisfying the diagnostic criteria for severe MDD. These symptoms caused significant social and occupational disability with her declining work only possible due to generous accommodations and financial need. She also reported alcohol consumption during adulthood in larger amounts or over a longer period than was intended, a strong desire to use, failure to fulfill major role obligations at home due to use, continued use despite having persistent social or interpersonal problems, increased activity participation in order to use, continued use despite knowledge of MDD that is likely to be exacerbated by use, and tolerance satisfying the diagnostic criteria for severe AUD. She denies additional substance use but reported a history of AUD in her father.

On exam, she was found to appear older than stated age, have no signs of acute distress besides occasional tears, low mood, dull affect, and mood congruence, some cognitive slowing, and thought content free of suicidal or homicidal ideation. In the past, she reportedly tried psychotherapy, paroxetine, bupropion, and another drug she could not remember (likely levomilnacipran) without significant improvement and was unable to consider repetitive transcranial magnetic stimulation because of work constraints on time.

**Figure 1:**
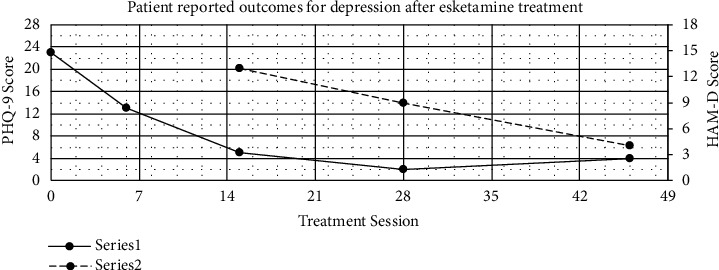
Patient reported outcomes for depression after Esketamine treatment. Over the course of treatment, the patient reported decreases in PHQ-9 and HAM-D scores. Series 1 indicates the PHQ-9 and series 2 indicates the HAM-D. Both demonstrate significant decreases in disease severity.

The patient was informed of the potential risks and benefits to esketamine therapy before consenting for treatment. While continuing 30 mg of oral paroxetine daily, she completed 20 sessions of 56 mg intranasal esketamine twice a week before increasing to 5 sessions weekly and then 21 sessions of 84 mg weekly to date. To date, she has exhibited no signs of tolerance or dependence to the esketamine. Although the patient reported immediate improvement in mood, the dosage was increased to sustain therapeutic effects throughout the entire intertreatment interval. She was able to complete these sessions with significant improvement determined subjectively and by validated outcomes measures for depression (Patient Health Questionnaire-9 [PHQ-9] and Hamilton Depression Rating Scale [HAM-D]) [13, 14]. From the start to the 46^th^ session, the patient's PHQ-9 scores fell from 23 to 4 indicating a decline from severe to none-minimal (Figure 1). From the 15^th^ session to the 46^th^, the patient's HAM-D scores fell from 13 to 4 indicating a decline from mild-to-moderate to mild. She also reported an improvement in anxiety, greater enjoyment in the relationships with her friends and family, resolution of her passive death wish, and the ability to continue her work as a registered nurse. This has all been accomplished without incidence of any adverse effects. There were initial clinically insignificant increases in systolic blood pressure of 10-15 mmHg that did not need adjustments to her hypertensive medications. She endorsed some high readings at home but attributes it to forgetting to take her medications.

Incidentally, she also reported the complete cessation of alcohol use by the second week due to decreased desire and impulsivity. To date, she has had a single episode of relapse with binge drinking while still on the lower dose of esketamine but after a single session of motivational therapy, has remained alcohol-free since. The plan is to continue esketamine at her current dose and frequency and then discuss the option of tapering down once she completes 6-8 months without a relapse of her depressive symptoms.

## 3. Discussion

This case presents the first documented example of the safe and effective use of intranasal esketamine in a patient with treatment resistant MDD in which there was incidental resolution of their comorbid severe AUD. Although its significance is limited by the characteristics of any case report (i.e., sample size, short-term data collection, and the lack of a control), the case documents an incidental finding of significant magnitude with relative simplicity and clarity. Due to the uncontrolled study design but chronology of disease onset and resolution, it would not be unreasonable to assume that the MDD may have been contributing to the AUD in this patient.

The mechanism of action for esketamine's antidepressant and possible alcohol cessation properties remain unclear but hypotheses include antagonism of the NMDA receptor, agonism of the AMPA receptor, activation of the anterior cingulate cortex, and increased connectivity between the insula and default mode network, and enhanced neuronal vascular endothelial growth factor signaling [15–18]. Despite a recent spike in interest for ketamine and its potential use in AUD, a 2022 review by Garel et al. was unable to fully support this claim [12]. However, they acknowledged that research is ongoing and suggested various study designs to further elucidate the potential relationship.

In conclusion, this case demonstrates the safe and rapidly effective therapeutic effects of esketamine in a patient with treatment resistant MDD which simultaneous resolution of comorbid AUD. Although there is insufficient evidence to support esketamine's direct potential role in treating AUD, perhaps this case shows that among patients with some subtypes of AUD in which there is an underlying MDD, resolution of the MDD is sufficient to significantly improve alcohol dependence. In addition to providing further evidence for countering fears of AUD as a contraindication, the case supports the call for greater access to esketamine therapy and additional studies designed to understand the mechanisms of esketamine and determine whether any esketamine intervention truly benefits patients with AUD.

## 4. Patient Perspective

“The next day after my first treatment I made a comment to my husband that it was a really nice day, and everything was brighter. Colors were richer. The sky was brighter. Literally, the world seemed brighter. After a few weeks, I was not sure if therapy was working. My husband had to share with my doctor that I was less angry. Our children had noticed a change in my behavior too. They were not yet aware that I have started any kind of therapy.

My anger has completely subsided. I never realized how I would get upset about things that did not matter. I would make things a big deal rather than just having an appropriate level of emotions for situations. I had completely stopped drinking alcohol. I had lost 35 pounds since stopping my daily consumption of alcohol. I think I am eating healthier because I am not searching for something to make me feel better. I feel 80% better. My children are aware of my treatment and are happy that I am taking steps to take care of myself, and they continue to notice improvements.”

The patient perspective was summarized with preservation of the patient's voice from their testimony.

## Data Availability

The data used to support the findings of this study are included within the article.
